# Pulmonary BRAF-driven Langerhans cell histiocytosis following selpercatinib use in metastatic medullary thyroid cancer

**DOI:** 10.1530/EDM-23-0079

**Published:** 2024-05-27

**Authors:** Katherine Wu, Shejil Kumar, Ed Hsiao, Ian Kerridge, Min Ru Qiu, Rhonda Siddall, Roderick Clifton-Bligh, Anthony J Gill, Matti L Gild

**Affiliations:** 1 Department of Diabetes, Endocrinology and Metabolism, Royal North Shore Hospital, Sydney, Australia; 2 Department of Radiology, Royal North Shore Hospital, Sydney, Australia; 3 Department of Haematology, Royal North Shore Hospital, Sydney, Australia; 4 Department of Anatomical Pathology, SydPath, St Vincent’s Hospital, Sydney, Australia; 5 St Vincent’s Clinical School, University of New South Wales, Sydney, Australia; 6 Cancer Genetics Unit, Kolling Institute of Medical Research, Sydney, Australia; 7 Northern Clinical School, University of Sydney Faculty of Medicine and Health, Sydney, Australia; 8 Department of Anatomical Pathology, Royal North Shore Hospital, Sydney, Australia; 9 Cancer Diagnosis and Pathology Group, Kolling Institute of Medical Research, Sydney, Australia

**Keywords:** Adult, Female, White, Australia, Thyroid, Endocrine-related cancer, Thyroid, Tumours and neoplasia, Genetics and mutation, Unusual effects of medical treatment, May, 2024

## Abstract

**Summary:**

*RET* mutations are implicated in 60% of medullary thyroid cancer (MTC) cases. The RET-selective tyrosine kinase inhibitor selpercatinib is associated with unprecedented efficacy compared to previous multi-kinase treatments. Langerhans cell histiocytosis (LCH) is a clonal histiocytic neoplasm usually driven by somatic *BRAF* mutations, resulting in dysregulated MAPK signalling. We describe a 22-year-old woman with metastatic MTC to regional lymph nodes, lung and liver. Tumour tissue harboured a somatic pathogenic *RET* variant *p.(M918T)* and selpercatinib was commenced. She experienced sustained clinical, biochemical and radiological responses. Two years later, she developed rapidly progressive apical lung nodules, prompting biopsy. Histopathology demonstrated LCH with a rare *BRAF* variant *p.(V600_K601>D)*. The lung nodules improved with inhaled corticosteroids. We hypothesize that selective pressure from RET blockade may have activated a downstream somatic *BRAF* mutation, resulting in pulmonary LCH. We recommend continued vigilance for neoplasms driven by dysregulated downstream MAPK signalling in patients undergoing selective RET inhibition.

**Learning points:**

- Patients with *RET*-altered MTC can experience rapid disease improvement and sustained disease stability with selective RET blockade (selpercatinib).
- LCH is a clonal neoplasm driven by MAPK activation, for which the most common mechanism is *BRAF* mutation.
- Both MTC and pulmonary LCH are driven by dysregulated MAPK signalling pathway activation.
- We hypothesise that the RET-specific inhibitor selpercatinib may have caused the activation of dormant LCH secondary to selective pressure and clonal proliferation.

## Background

The majority (60%) of medullary thyroid cancers (MTCs) are driven by mutations in the rearranged during transfection (*RET*) proto-oncogene, leading to inappropriate activation of growth factor signalling pathways such as the mitogen-activated protein kinase (MAPK) pathway (1). The RET-downstream kinase activation cascade includes RAS, followed by BRAF, MEK and ERK, culminating in dysregulated gene expression in genes involved with cell survival and proliferation, ultimately resulting in tumorigenesis (2, 3). Non-selective multi-kinase inhibitors such as cabozantinib and vandetanib are approved for the treatment of MTC, with inhibition extending beyond RET to other kinases such as vascular endothelial growth factor (VEGF) and epidermal growth factor (EGF). However, ‘off-target’ effects often result in substantial and intolerable adverse effects such as diarrhoea, hypertension and palmar-plantar erythrodysesthesia syndrome, necessitating treatment interruption or dose reduction in many patients (4).

Selpercatinib is a highly selective, small molecule RET kinase inhibitor. In the LIBRETTO-001 phase I/II trial, selpercatinib demonstrated unprecedented efficacy and safety in treating various RET-altered advanced malignancies, including MTC, with greater disease response, progression-free survival and fewer toxicities compared to results with multi-kinase inhibitors in other trials (5). Phase III trials are currently underway, comparing progression-free survival of selpercatinib to the current standard of care: cabozantinib or vandetanib.

Langerhans cell histiocytosis (LCH) is a clonal neoplasm of myeloid dendritic cells, unrelated to MTC. It is usually driven by somatic mutations in *BRAF* or other genes in the MAPK pathway downstream of RET, resulting in dysregulated activation of this pathway and uncontrolled cell growth (3).

We present an unusual novel case of BRAF-driven pulmonary LCH occurring following prolonged RET inhibition with selpercatinib. We propose that selective pressure from RET blockade promoted mutational activation of downstream BRAF kinase signalling and may have contributed to the development of LCH. The study was approved by the Northern Sydney Local Health District Human Research Ethics Committee, which includes a waiver of consent policy for the use of archived formalin-fixed, paraffin-embedded tissue (2019/ETH08420). Informed consent was obtained from the patient for the publication of this case report.

## Case presentation

A 22-year-old woman presented in October 2019 with 6 months of profuse diarrhoea, night sweats, hot flushes, dysphagia and 20 kg unintentional weight loss. Recent gastroscopy and colonoscopy were unremarkable. She had no significant past medical or family history, was not taking any regular medications and was a 5-pack year ex-smoker.

## Investigation

**Figure 1:**
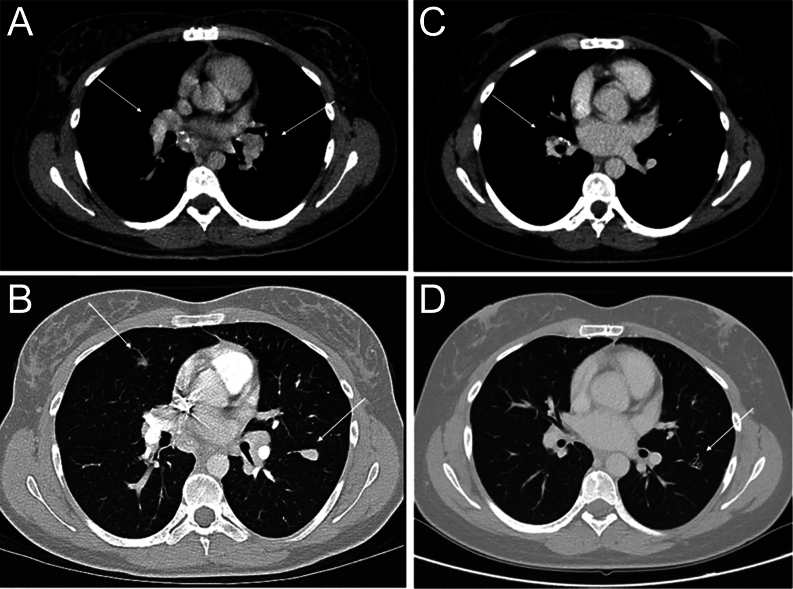
Serial CT scans showing radiological response to selpercatinib. Axial-view CT scan of the chest demonstrates bulky bilateral hilar lymphadenopathy (A) and bilateral pulmonary nodules (B) and subsequent radiological response (C, D) 3 months after commencing selpercatinib.

CT scan of the chest, abdomen and pelvis demonstrated bulky mediastinal and hilar lymphadenopathy (20 mm), multiple pulmonary nodules (dominant lesion 14 mm) and multiple liver lesions (Fig. 1). Calcified lesions on the left side of the neck were suspicious for a thyroid lesion and adjacent lymphadenopathy. Thyroid ultrasound revealed a 36 mm hypoechoic lesion in the left thyroid gland with enlarged cervical lymphadenopathy. Results of fine needle aspiration biopsy of the thyroid lesion suggested MTC. Serum calcitonin concentration was markedly elevated at 25 600 ng/L (normal range <20).

A dedicated CT scan of the neck demonstrated a left lower thyroid lesion with internal calcification and enlarged lymph nodes with internal calcifications (largest 24 mm). The right thyroid lobe appeared unremarkable. MRI scan of the liver with contrast showed multiple enhancing liver lesions. MRI brain scan was unremarkable. ^68^Ga-DOTATATE-PET/CT scan showed increased avidity in the left thyroid lobe, bilateral supraclavicular, hilar and mediastinal nodes, bilateral lung nodules and liver lesions.

Biochemical screening for other multiple endocrine neoplasia (MEN)-2 associated conditions was negative, including corrected calcium 2.38 mmol/L (2.10–2.60), plasma normetanephrines 628 pmol/L (<560) and metanephrines 104 pmol/L (<447). Germline DNA sequencing did not reveal pathogenic RET variants. She underwent a two-staged debulking procedure: (1) total thyroidectomy and left-sided neck dissection and (2) sternotomy and mediastinal/hilar lymph node resection. Histopathology confirmed unifocal left-sided MTC (45 mm) with 23/82 positive neck lymph nodes (largest 29 mm), 8/10 positive mediastinal/hilar lymph nodes (largest 20 mm), positive surgical margins, vascular invasion and a Ki67 proliferative index of 2%. Tumour tissue DNA sequencing revealed a heterozygous somatic pathogenic variant *p.(Met918Thr)* in exon 16 of the *RET* gene.

## Treatment

She subsequently commenced 160 mg BD selpercatinib, a selective RET tyrosine kinase inhibitor (TKI) for metastatic sporadic *RET*-mutated MTC as part of the LIBRETTO-001 phase II clinical trial in March 2020. She experienced rapid symptomatic (resolution of diarrhoea and weight re-gain), biochemical (declining calcitonin concentrations to 56 ng/L) and radiological improvement (interval reduction in size of target lesions), with partial response as per RECIST 1.1 criteria (Fig. 1). She had stable disease for 2 years after commencing selpercatinib with a minor adverse effect of small bowel oedema manifesting as abdominal pain requiring a 2-week interruption, 18 months after commencing therapy.

### Outcome and follow-up

**Figure 2:**
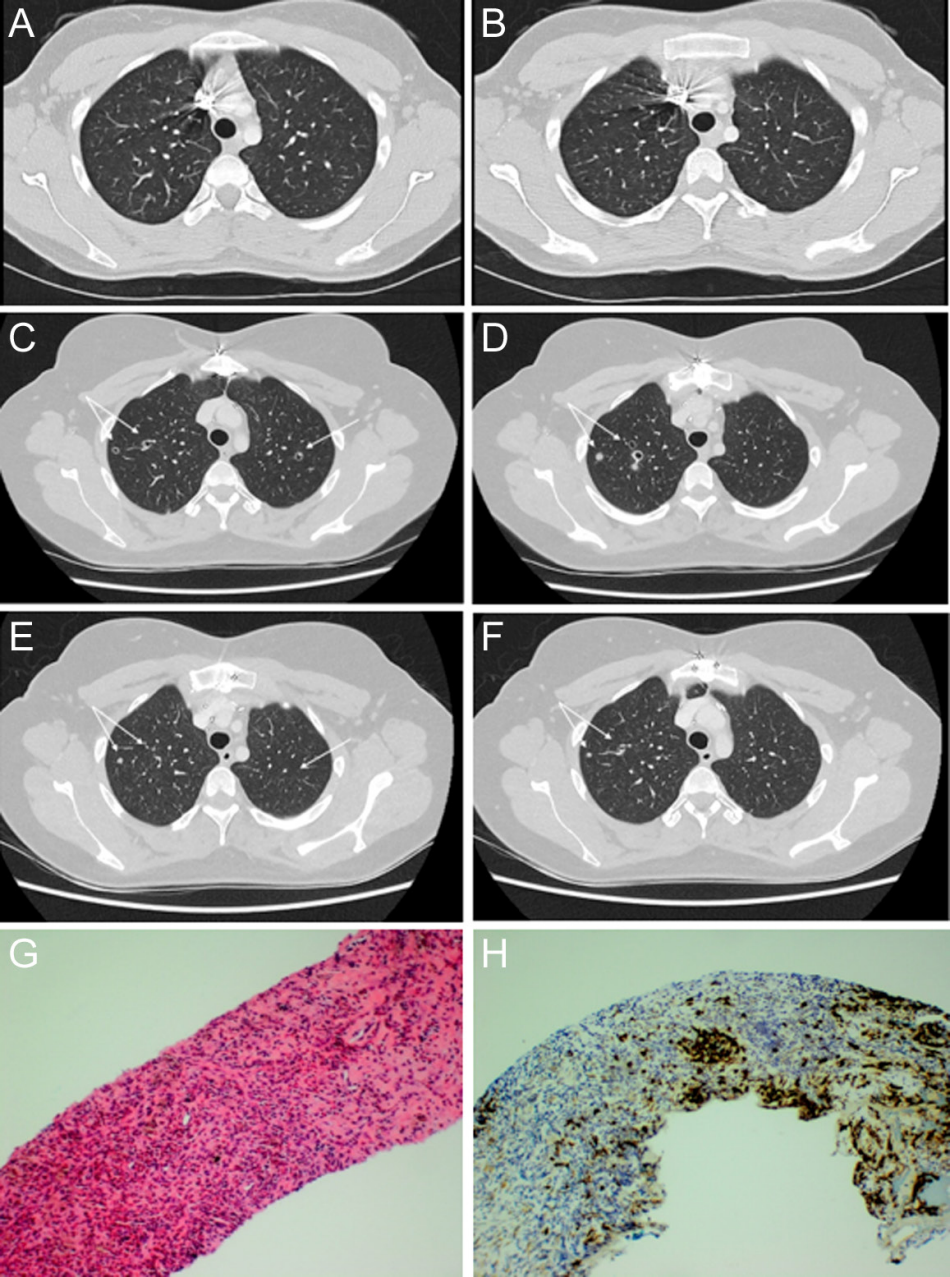
Cavitating apical lung nodules pre- and post-inhaled corticosteroids and surgical pathology of the lung biopsy. Axial-view CT scan of the chest demonstrates bilateral cavitating subcentimetric apical nodules (C, D) which were not present on the initial CT scan pre-selpercatinib (A, B). There was almost complete resolution 3 months later with inhaled glucocorticoids (E, F). Serial haematoxylin and eosin (H&E) (G) and Langerin immunohistochemistry (IHC) (H) stained sections of the lung core biopsy demonstrating Langerhans cell histiocytosis (LCH). (G) On H&E, the Langerhans cells are visible but obscured by an infiltrate of chronic inflammatory cells rich in eosinophils. (H) Langerin IHC highlights the Langerhans cells which account for about 5% of the cells in the section (original magnification 100×).

In March 2022, new 4 mm bilateral apical pulmonary nodules were noted on a progress CT chest scan which rapidly enlarged to 8 mm the following month and exhibited a cavitating appearance, in association with 2 months of dry cough (Fig. 2). ^18^F-FDG-PET/CT scan demonstrated low avidity (SUVmax 3.5). Concerns for acquired resistance to selpercatinib and progressive MTC led to diagnostic CT-guided lung biopsy which revealed a diffuse inflammatory infiltrate including chronic inflammatory cells rich in eosinophils with less abundant Langerhans type histocytes (Fig. 2). No pathogenic organisms were identified with special stains for fungi and AFB and no evidence of MTC was identified by morphology or with immunohistochemistry for calcitonin. Specific immunohistochemistry confirmed that the histiocytes were Langerhans cells (CD1a, S100, Langerin (CD207) positive] (Fig. 2). Next-generation sequencing testing by using Oncomine Precision Assay on Genexus system was negative for the classic *BRAFV600E* mutation but demonstrated the rare complex *BRAF* mutation *p.(V600_K601>D)* at a variant allele frequency of 4.76% in keeping with the expected neoplastic cellularity. Although not previously described in LCH, this variant has been reported to be pathogenic in melanoma (6).

A diagnosis of BRAF-driven pulmonary LCH was made. She was commenced on inhaled corticosteroids with subsequent reduction in the size of her lung nodules (Fig. 2). Her MTC disease status has remained stable 3 years since commencing selpercatinib (calcitonin ranging between 21 and 42 ng/L), and she will have ongoing close follow-up in a multidisciplinary team setting.

## Discussion

While the efficacy of selpercatinib in advanced RET-altered malignancy, including MTC, has been well demonstrated in recent clinical trials, long-term outcomes and potential adverse complications have yet to be established. To our knowledge, this is the first reported case demonstrating a patient with pulmonary BRAF-driven LCH occurring after prolonged RET inhibition by selpercatinib. Here, we explore the potential risk of developing other MAPK-driven neoplasms secondary to the activation of mutations downstream of selective RET inhibition.

RET is a transmembrane receptor tyrosine kinase that, upon activation, propagates downstream signalling in several growth factor signalling pathways, including the MAPK pathway (2). This triggers a phosphorylation cascade of kinases RAS, BRAF, MEK and then ERK, ultimately leading to changes in the expression of genes promoting cell differentiation, proliferation, and survival (2, 3). RET alteration fosters unregulated activation of RET signalling, driving oncogenesis in MTC, and thus treatment depends on the use of multi-kinase inhibitors (exhibiting non-selective RET blockade) or selective RET TKIs such as selpercatinib.

Reactivation of MAPK signalling pathways after RET-blockade with TKIs, including selpercatinib, is a well-described phenomenon, resulting in the potential for TKI resistance and cancer progression. Rosen *et al.* described two potential mechanisms of selpercatinib resistance driven by reactivation of the MAPK pathway: (1) due to ‘on target’ secondary *RET* mutations preventing binding of selpercatinib to RET and (2) ‘off target’ mutations in downstream MAPK signalling pathways that bypass RET-blockade (7). Rosen *et al.* included two patients with *RET* mutations who developed acquired resistance to selpercatinib. At the time of disease progression, there was a decrease in the frequency of *RET* alterations but a converse increase in the frequency of a *KRAS*-mutant allele. This suggested the *RET* fusion and *KRAS* mutation were likely not present in the same cell population but selpercatinib created a selective pressure, while eliminating the sensitive *RET* fusion-positive cells, fostering emergence of a resistant *KRAS* mutant cell population. Furthermore, after monotherapy with BRAF inhibitors was first trialled in melanoma, the eruptive development of other MAPK-driven neoplasms (most commonly *RAS* mutant keratoacanthomas and cutaneous squamous cell carcinomas) was reported (8). This was attributed to the TKI causing a paradoxical increase in MAPK signalling in other cells harbouring pre-existing *RAS* mutations. Although the mechanism underpinning this paradoxical effect is not entirely clear, it is potentially related to a conformational change in wild-type BRAF protein, CRAF dimerisation and ERK activation, each independent of RAS activation (7). Thus, this supports the concept that despite selective kinase inhibition, activation of various other drivers along the MAPK pathway is still possible and can result in secondary neoplasms.

LCH is a clonal neoplasm of myeloid dendritic cells expressing a Langerhans cell phenotype (CD1a and CD207 expression). Isolated pulmonary LCH predominantly occurs in young adult smokers and most commonly presents with cough and exertional dyspnoea. Occurrence of LCH has been reported in association with other malignant neoplasms, most commonly haematological. An international registry identified a young cohort of 54 patients with LCH with associated tumours including acute leukaemia (*n* = 29), lymphoma (*n* = 4) and various solid tumours (*n* = 21) in which the majority of these malignancies occurred after chemoradiotherapy for LCH (9). In older patients, the co-existence of lung cancer and pulmonary LCH has also been found, potentially reflecting a local Langerhans cell reaction to the tumour (10). Three cases have been described of the same oncogenic MAPK mutation (e.g. *NRAS*, *KRAS*) driving both LCH and additional haematological malignancies in the same patient (11). LCH has been reported to occur in two cases secondary to malignant melanoma treatment, although *BRAF* sequencing was not performed (12).

LCH is unrelated to MTC but is also driven by alterations and subsequent activation of the MAPK pathway, most commonly the *BRAFV600E* mutation, which is present in more than half of LCH cases (3). In the case described, we propose that RET selective inhibition with selpercatinib may have resulted in the selection for a *BRAF*-mutated pulmonary Langerhans cell population, resulting in activation and rapid progression of an otherwise dormant LCH. The ongoing response to selpercatinib and lack of acquired resistance in our patient suggest downstream MAPK signalling activation in MTC cells has not occurred.

In investigating alternative mechanisms, we examined the possibility that histiocytes may express RET and therefore may be susceptible to downstream MAPK signalling pathway activation in response to selpercatinib-induced RET inhibition. Although literature exploring histiocyte expression of RET is scarce, one study evaluated RET expression by immunohistochemistry in 50 cases of papillary thyroid cancer and found that in 26/50 (52%), adjacent histiocytes stained positive for RET (13). In most cases, the histiocytes were found within the inflammatory infiltrate of the tumour. None of these patients had chronic autoimmune lymphocytic thyroiditis. Furthermore, Durham *et al.* conducted somatic genomic analysis on histiocytic tissue biopsies obtained from 270 patients with various histiocytic disorders (14). One patient with *RET*-rearranged disseminated cutaneous xanthogranuloma was treated with selpercatinib resulting in dramatic resolution of lesions. Hence, limited data suggest that histiocytes may express RET, leading to the possibility of activating downstream MAPK mutations in response to RET inhibition and the development of secondary histiocytic disorders as described.

To date, there have been no other reported cases of LCH or other MAPK pathway-driven neoplasms arising in patients undergoing prolonged RET inhibition. We recommend continued vigilance for the possibility that RET inhibition may activate other underlying neoplasms with downstream mutations in the MAPK pathway.

## Declaration of interest

The authors declare that there is no conflict of interest that could be perceived as prejudicing the impartiality of this case report.

## Funding

This study did not receive any specific grant from any funding agency in the public, commercial or not-for-profit sector.

## Patient consent

Written informed consent for publication of their clinical details and clinical images was obtained from the patient.

## Author contributions

KW assisted with acquisition of data and drafted the original manuscript. SK acquired data, assisted with conceptualisation, drafted the original manuscript and critically reviewed the manuscript. EH assisted with conceptualisation and critically reviewed the manuscript. IK assisted in management of the patient, conceptualisation and critically reviewed the manuscript. MQ assisted with conceptualisation and critically reviewed the manuscript. RS assisted with conceptualisation and critically reviewed the manuscript. RC assisted with conceptualisation and critically reviewed the manuscript. AG assisted in specialised pathological investigation, conceptualisation and critically reviewed the manuscript. MG managed the patient, conceptualised the manuscript and critically reviewed the manuscript. All authors (KW, SK, EH, IK, MQ, RS, RC, AG, MG) approved the final version of the manuscript and agreed to be accountable for all aspects of the work.
